# Impact of the COVID-19 pandemic on patients with coronary artery disease requiring cardiac surgery at a German university hospital

**DOI:** 10.1186/s13019-025-03373-2

**Published:** 2025-02-15

**Authors:** Jan S. Englbrecht, Jan K. Landwehrt, Henryk Welp, Sven Martens, Antje Gottschalk

**Affiliations:** 1 Department of Anesthesiology, Intensive Care Medicine and Pain Therapy, University Hospital Münster, Albert-Schweitzer-Campus 1, Building A1, 48149 Münster, Germany; 2 Department of Cardiothoracic Surgery, University Hospital Münster, Münster, Germany; 3 Department of Anesthesiology, Intensive care and pain therapy, Florence-Nightingale-Hospital, Düsseldorf, Germany

**Keywords:** COVID-19 pandemic, Lockdown, Coronary artery disease, Cardiac surgery, Mortality

## Abstract

**Background:**

Studies show conflicting results regarding the impact of the COVID-19 pandemic on the treatment of patients with coronary artery disease requiring cardiac surgery and data from Germany are lacking. In this study, two patient cohorts who underwent coronary artery bypass graft surgery before and after the start of the COVID-19 pandemic were compared.

**Methods:**

Patients who presented for coronary artery bypass graft surgery before (01.05.18–30.04.19; group “B”) or during the COVID-19 pandemic (01.05.20-30.04.21; group “P”) at the University Hospital Münster in Germany were retrospectively identified and compared regarding demographics, preoperative status, surgical data, and postoperative outcome.

**Results:**

513 (group “B”) and 501 patients (group “P”) were included, demographics were comparable. In group “P”, preoperative myocardial infarction and emergency indications were more frequent, heart-lung machine and aortic clamping times were longer. Postoperative ICU-days and inpatient stay did not differ. Postoperative need of an extracorporeal life support system and intrahospital mortality tended to be higher in group “P”, without reaching statistical significance.

**Conclusion:**

The COVID-19 pandemic had a significant impact on cardiac surgical care with the prioritization of emergency procedures. Patients treated during the pandemic were in a more critical preoperative condition, duration of surgery was longer, but post-operative mortality was comparable.

## Introduction

Healthcare services were restricted worldwide during the COVID-19 pandemic, especially at the beginning of the pandemic situation. In Germany, this included a significant decrease in time-critical interventions for example in oncology, emergency care, or the treatment of heart attacks and strokes [1, 2].

Coronary artery disease (CAD) is among the most relevant diseases in Germany [3]. In addition to optimizing predisposing risk factors, critical CAD often requires interventional myocardial revascularization or coronary artery bypass graft (CABG) surgery. Reduced healthcare capacity could have a dramatic impact on patient outcomes, especially in this patient population.

An analysis of the number of cardiovascular non-invasive and invasive diagnostic procedures from 108 countries showed a 64% decrease in 2020 compared to 2019 [4]. A meta-analysis of 27 international studies showed a significant reduction in hospital admissions of patients with myocardial infarction during the pandemic, but no increase in mortality [5]. Data from Italy, on the other hand, showed significantly reduced admissions of patients with acute myocardial infarction (minus 48%) as well as a significant increase in mortality after ST-elevation myocardial infarction [6].

On March 16, 2020, the first restrictions were announced in Germany as part of a pandemic-related lockdown [7]. As a result, cardiac surgery, cardio-anesthesiology and intensive care were negatively affected in two main ways: firstly, elective surgery was postponed during the lockdown [8–10] and secondly, the more or less pronounced use of intensive care unit (ICU) capacities for COVID-19 patients limited the possibility of cardiac surgical care [11, 12]. In Germany, CABG surgery was reduced by 14% in 2020 compared to 2019 [3]. There was also a significant decline in CABG surgery in 2020 in other countries [13–16]. The delayed treatment and consecutive progression of the underlying disease with potentially worsened preoperative conditions may have had a negative influence on postoperative outcomes [3, 17]. Differentiated analyses of healthcare during the pandemic are therefore required for future, evidence-based recommendations for prioritizing treatment in pandemic or comparable crisis situations [1]. Due to the lack of centralized data collection and evaluation of healthcare capacities in the German healthcare system, estimating the effects of the pandemic on the healthcare, morbidity and mortality of CAD patients are only possible with a delay, if at all [1].

This study analyzed whether restrictions due to the COVID-19 pandemic had an impact on the care of cardiac surgery patients at the University Hospital Münster (UKM). Two cohorts of patients with CAD requiring CABG surgery were compared in terms of their preoperative condition, perioperative course and postoperative outcomes over a period of one year before and one year after the start of the first lockdown restrictions. With reference to results from international studies, we hypothesize that fewer patients could be treated during the pandemic due to limited resources, that they were in a more critical preoperative condition and had inferior postoperative outcomes.

## Methods

This retrospective study was performed in accordance with the Declaration of Helsinki. The need for informed consent was waived by the local Ethics Committee of the University of Münster due to the retrospective analysis of routinely collected patient data and the study protocol was approved on November 5th, 2021 (file number 2021-681-f-S). This was an exploratory study and not based on a formal power analysis [18]. As part of the database analysis, the clinical data of all patients with CABG surgery at the University Hospital Münster in Germany were retrospectively assessed. Two groups were examined, each over a period of one year. Firstly, patients who underwent surgery before the COVID-19 pandemic (period 01.05.18–30.04.19; group “B”– **b**efore pandemic), and secondly, patients who underwent cardiac surgery after the start of the first lockdown (period 01.05.20–30.04.21; group “P”– **d**uring pandemic). The individual medical record files were reviewed to obtain a complete data set for each identified patient.

Inclusion criteria were defined as:

- Patients ≥ 18 years.
- CABG surgery.

Exclusion criteria were defined as:

- combination surgery (CABG surgery plus valve replacement).
- patients with acute endocarditis.
- patients undergoing implantation of a left ventricular assist device (LVAD).
- CABG surgery after complications during interventional myocardial revascularization or after trauma.
- proven COVID-19 infection during inpatient stay.

To describe the population, the basic demographic data of both groups were recorded (age on admission, sex, height, weight, body mass index, distance from hometown to UKM). With regard to preoperative conditions, the presence of a preoperative myocardial infarction, the ASA risk classification (ASA - American Society of Anesthesiologists), the preoperative left ventricular ejection fraction (LVEF), the log EuroSCORE I (a scoring system designed to predict a patient’s mortality during cardiac surgery [19]) and the number of coronary arteries affected were recorded. Additionally, preoperative hemoglobin, creatinine and cardiovascular preoperative medication was assessed. Perioperative data included type of indication (elective, urgent, emergency surgery), inhospital resuscitation preoperatively or preoperative use of an extracorporeal life support (ECLS) system, heart-lung machine (HLM) time, aortic clamping time during surgery and the share of procedures using Off-pump coronary artery bypass graft (OPCAB) technique. The outcome parameters recorded were the length of stay (LOS) in the ICU and total inpatient LOS, an intraoperatively implemented ECLS system and mortality during the inpatient stay.

The change in ICU treatment capacities at the UKM during the pandemic was assessed as previously described [20]. Briefly, the average ICU occupancy days (ICU-OD, defined as the sum of the fully inpatient ICU-patients of each day at 12 p.m. (sum of midnight stock)) were assessed for the year 2019 and 2020 at the UKM. Information about ICU-OD was taken from internal quality reports.

### Statistical methods

Statistical analysis was performed using SPSS (IBM, version 28). The data evaluation included descriptive and analytical statistics. The selection of statistical tests depended on the type of data. The independent t-test was used to compare the monthly numbers of CABG surgery. A one-way multivariate analysis of variance (MANOVA) was used to compare the groups “B” and “P”, and predefined dependent ordinal or interval scaled variables (age on admission, height, weight, body mass index, distance from hometown to UKM, American Society of Anesthesiologists (ASA) risk classification, left ventricular ejection fraction (LVEF), log EuroSCORE I, number of affected coronary arteries, indication, HLM-time, aortic clamping time, ICU length of stay, total inpatient LOS). Post-hoc test for each dependent variable was performed using a one-way ANOVA, if indicated. In case of nominal scaled dependent variables (sex, preoperative myocardial infarction, ECLS system, inhouse mortality), the Chi-Square test of independence was used. A binomial logistic regression was performed to determine the effect of postoperative ECLS therapy, indication for CABG, and surgery during corona pandemic to predict the likelihood of mortality. Additionally, a Kaplan-Meier survival curve was created to analyze and visualize the ’time-to-inhospital death’ data. A *p*-value ≤ 0.05 was defined as statistically significant. *P*-values of all post-hoc ANOVA and Chi-square test were adjusted with the Holm-Bonferroni method due to multiple (seven-fold) testing.

## Results

A total of 521 patients were identified in group “B” (period 01.05.18–30.04.19; **b**efore pandemic) and 510 patients in group “P” (period 01.05.20–30.04.21; **d**uring pandemic), respectively. In group “B”, three patients were excluded due to endocarditis and five due to CABG surgery following complications (after interventional myocardial revascularization or trauma). In group “P”, one patient was excluded due to CABG surgery after trauma and eight patients due to a proven COVID-19 infection during their inpatient stay. This resulted in 513 patients in group “B” and 501 patients in group “P” for further analysis, respectively. Information on all predefined variables could be obtained from each of the included patients. Both groups were independent, no patient was included in both groups.

### Number of CABG surgeries

**Fig. 1:**
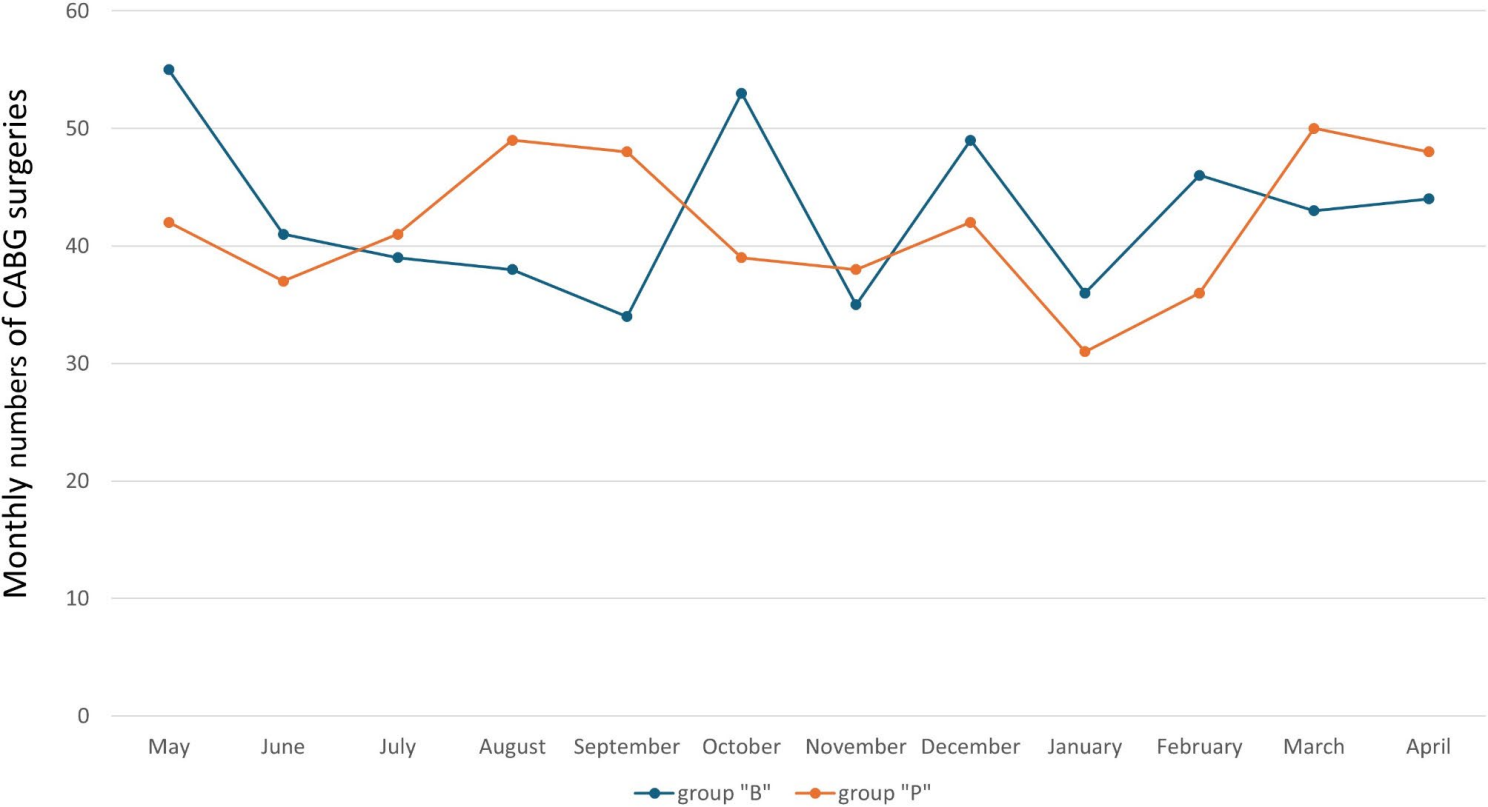
Monthly coronary artery bypass graft surgeries at the University Hospital Münster. Group “B” (01.05.2018–30.04.2019), before the COVID-19 pandemic, and group “P” (01.05.2020–30.04.2021), during the COVID-19 pandemic

Overall, there was a 2.3% decrease in the number of CABG surgeries performed. The number of CABG surgeries per month did not differ significantly between both groups (“B”/”P”, mean ± standard deviation: 42.8 ± 6.9 / 41.8 ± 6.0, *p* = 0.709; Fig. 1).

### Demographics

There was no statistically significant difference between group “B” and “P” for the demographic variables (Table 1).

**Table 1:** Preoperative demographics and characteristics of the study cohorts

Variable	Group B	Group *P*	*p*
Included patients [n]	513	501	0.254
Age (years, mean ± SD)	68.2 ± 9.8	67.9 ± 9.7	
Height (cm, mean ± SD)	173.7 ± 8.9	174.5 ± 8.4	
Weight (kg, mean ± SD)	84.7 ± 17.2	86.1 ± 16.4	
Body mass index (kg/m², mean ± SD)	28.0 ± 5.0	28.2 ± 4.8	
Distance hometown to UKM (km, mean ± SD)	56.1 ± 60.8	51.6 ± 50.6	
Sex [n] (male/female)	412/101 80%/20%	404/97 81%/19%	0.896*
Preoperative myocardial infarction [n]	108 (21%)	238 (48%)	0.007*
ASA risk classification [n] (1/2/3/4/5)	0/47/429/35/2 0%/9%/84%/7%/0.3%	1/56/404/40/0 0.2%/11%/81%/8%/0%	0.676
LVEF [n] (≥ 50%/31–50%/21–30%/≤ 20%)	372/96/30/15 73%/19%/6%/3%	352/116/23/10 70%/23%/5%/2%	
Log EuroSCORE I [n] (mean ± SD)	6.8 ± 8.7	7.4 ± 10.1	
CAD [n] (one-/two-/three-vessel)	14/76/423 3%/15%/82%	10/85/406 2%/17%/81%	

Group B: period from 01.05.18 to 30.04.19; group P: period from 01.05.20 to 30.04.21; SD: standard deviation; UKM: University Hospital Münster, ASA: American Society of Anesthesiologists; LVEF: left ventricular ejection fraction; CAD: coronary artery disease; *Holm-Bonferroni adjusted *p*-values

### Preoperative characteristics

Overall incidence of a preoperative myocardial infarction was significantly higher in group “P” (Table 1). A total of 86 patients had an infarction within the last three weeks prior to CABG surgery (group “B” / group “P”: *n* = 8/*n* = 78), and 143 patients within 48 h prior to surgery (group “B” / group “P”: *n* = 38/*n* = 105), respectively. All other preoperative characteristics showed no significant differences between both groups (Table 1). Preoperative laboratory values and cardiovascular preoperative medications were also comparable (Table 2).

**Table 2:** Preoperative laboratory values and medications

Laboratory values/medication	Group B (*n* = 513)	Group P (*n* = 501)
Hemoglobin (g/dl, mean ± SD]	13.45 ± 1.76	13.53 ± 1.93
Creatinine (mg/dl, mean ± SD]	1.14 ± 0.83	1.15 ± 0.82
Aspirin [n]	414 (81%)	395 (79%)
Clopidogrel [n]	62 (12%)	59 (12%)
Digitalis [n]	8 (2%)	4 (1%)
Diuretics [n]	180 (35%)	173 (35%)
Beta blockers [n]	327 (64%)	286 (57%)
Calcium antagonist [n]	137 (27%)	163 (33%)
Angiotensin-converting enzyme-inhibitors [n]	344 (67%)	367 (73%)
Phenprocoumon [n]	22 (4%)	14 (3%)

Group B: period from 01.05.18 to 30.04.19; group P: period from 01.05.20 to 30.04.21

### Surgical data

Comparing the combined dependent surgical data showed significant differences (F [3, 1010] = 7.854, *p* < 0.001, partial η² = 0.023, Wilk’s Λ = 0.977). Post-hoc tests revealed a significantly different distribution of indications. In group “P”, procedures were more often classified as urgent or emergency surgery. Intraoperative HLM-times and aortic clamping times were significantly longer in group “P” (Table 3). The OPCAB technique was used in 4 cases in group “B” and in 6 cases in group “P”. Only in group “P” were patients who were resuscitated (*n* = 4) or needed an ECLS system preoperatively (*n* = 4).

**Table 3:** Surgical data of the study cohorts

Variable	Group B (*n* = 513)	Group P (*n* = 501)	*p*
Indication [n] (elective/urgent/ emergency)	265/185/63 52%/36%/12%	223/192/86 45%/38%/17%	0.032*
HLM-time (minutes ± SD)	99 ± 32	107 ± 35	0.007*
Aortic clamping time (minutes ± SD)	58 ± 21	64 ± 24	0.007*

Group B: period from 01.05.18 to 30.04.19; group P: period from 01.05.20 to 30.04.21; HLM: heart-lung machine; SD: standard deviation; CABG: coronary artery bypass graft; AVR: aortic valve replacement; *Holm-Bonferroni adjusted *p*-values

### Outcome data

The assessed outcome data did not differ significantly between group “B” and “P”. The number of patients with an implemented ECLS system intraoperatively and the inpatient mortality was higher in group “P”, without reaching statistical significance (Table 4).

**Table 4:** Outcome data of the study cohorts

Variable	Group B (*n* = 513)	Group P (*n* = 501)	*p*
ICU length of stay (days ± SD)	2.2 ± 5.2	2.5 ± 4.9	0.600
Length of hospital stay (days ± SD)	13.0 ± 10.8	13.2 ± 12.8	
ECLS post CABG surgery [n]	10 (1.9%)	17 (3.4%)	0.459*
Mortality during hospital stay [n]	16 (3.1%)	24 (4.8%)	0.459*

Group B: period from 01.05.18 to 30.04.19; group P: period from 01.05.20 to 30.04.21; SD: standard deviation; ICU: intensive care unit; ECLS: Extracorporeal Life Support; CABG: coronary artery bypass graft; *Holm-Bonferroni adjusted *p*-values

The binomial logistic regression model was statistically significant, χ²(3) = 43.421, *p* < 0.001. Of the three variables that entered the regression model, two contributed significantly to predict mortality: ECLS post CABG surgery (*p* < 0.001) and indication for CABG surgery (*p* = 0.004), while classification to group B or group P showed no significant effect (*p* = 0.415). Perioperative implementation of an ECLS system increased the likelihood of mortality, OR = 12.655 (95%-CI [5.222, 30.671]), as did urgent or emergency indication for CABG, OR = 3.429 (95%-CI [1.466, 8.021]).

### Kaplan Meier survival curve

**Fig. 2:**
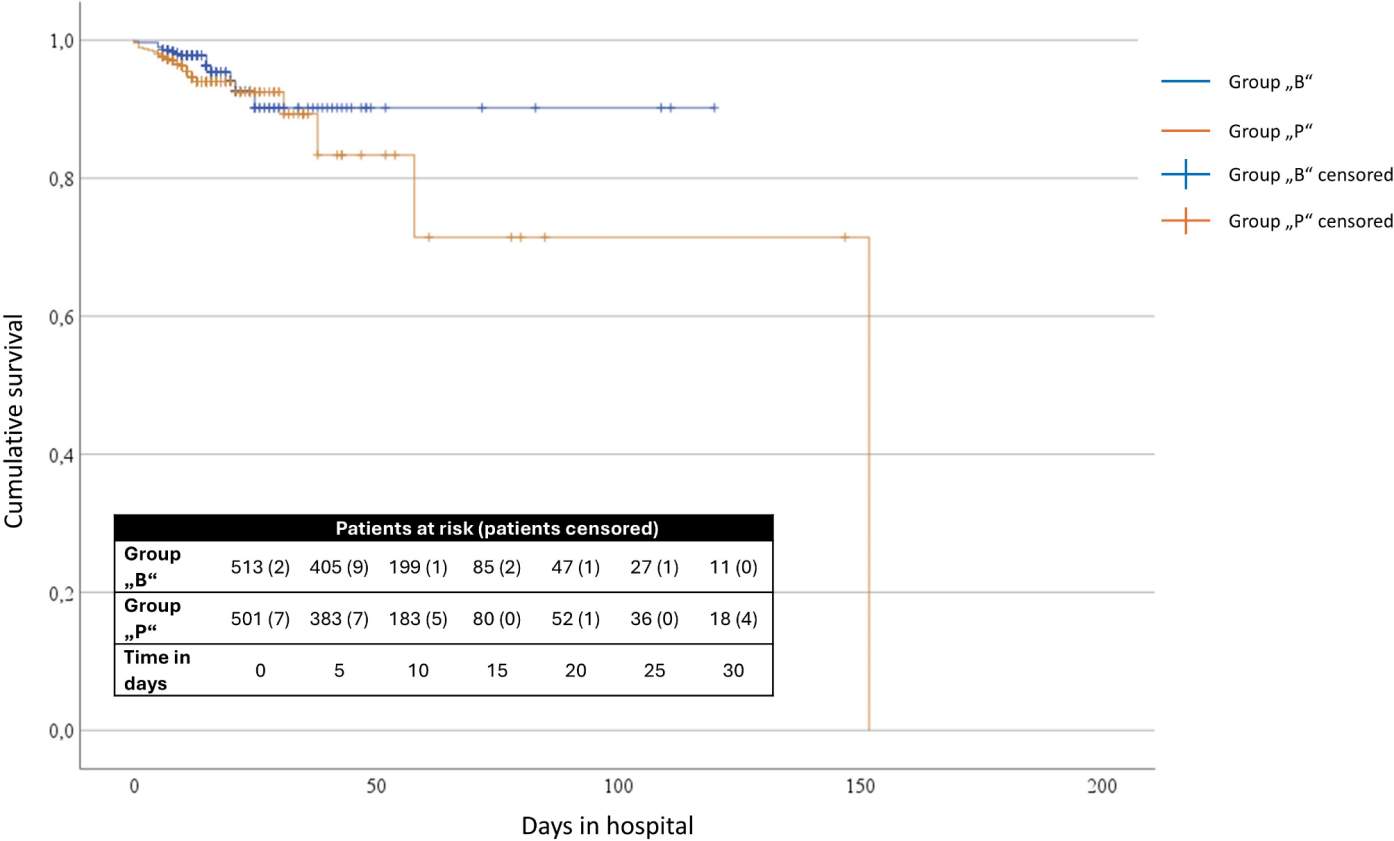
Kaplan Meier survival curve of the included patients. Before (group “B”: 01.05.2018–30.04.2019) and during (group “P”: 01.05.2020–30.04.2021) the COVID-19 pandemic

A log-rank test was performed to assess whether significant differences existed between the two groups regarding inhospital mortality. Results showed that mortality was higher in group “P”, but survival distributions did not differ significantly, χ² (1) = 1.287, *p* = 0.257 (Fig. 2).

### ICU treatment capacities

ICU-OD for the different ICU-disciplines at the UKM showed a decrease in treatment capacities for all disciplines in 2020 compared to 2019, with the strongest reduction in the non-operative and pediatric ICU-departments (Table 5).

**Table 5:** Average daily occupancy days in the various intensive care units

Type of ICU	2019	2020	Difference [%]
Pediatric [ICU-OD]	26.16	21.70	-17.0
Neurologic [ICU-OD]	9.89	9.66	-2.3
Non-operative [ICU-OD]	19.18	16.62	-13.3
Operative [ICU-OD]	38.62	37.50	-2.9
Total [ICU-OD]	93.85	85.48	-8.9

ICU-OD: Average intensive care occupancy days (sum of the fully inpatient ICU-patients of each day at 12 p.m.)

## Discussion

The number of monthly CABG surgeries at the UKM was only slightly reduced during the first year of the COVID-19 pandemic compared to the year before but was accompanied by a higher incidence of preoperative myocardial infarction and a shift towards more emergency indications, which was accompanied by a higher risk of inhospital death. Surgery times were longer during the pandemic. Postoperative days in the ICU and total inpatient LOS were comparable. The need for implanting an ECLS system intraoperatively and inpatient mortality was comparable in patients who underwent CABG surgery during the pandemic.

The incidence of preoperative myocardial infarction was significantly higher during the pandemic in our cohort, indicating that patients with CABG surgery during the pandemic were in a more advanced stage of the disease. Overall, CAD morbidity in Germany fell significantly in 2020 compared to 2018 (-11.4%) and 2019 (-12.3%). This applies to both the raw and the age- and gender-standardized hospitalization rates [3]. However, these data barely allow the conclusion that CAD morbidity per se was rarer during the pandemic. It is more likely, that the COVID-19-related restrictions led to an underdiagnose of CAD or reduced hospital admissions of CAD patients [3, 21]. This assumption is supported by results showing a significant overall decline in cardiac diagnostic procedures and an associated decline in diagnosed myocardial infarctions in 2020 both in Germany and worldwide at the beginning of the pandemic [4, 21–23]. Numerous studies provide explanations for this presumably pandemic-related phenomenon. Firstly, many patients avoided hospital admission for fear of COVID-19 infection [24–30]. Secondly, typical symptoms that actually indicate a cardiac problem may have been misinterpreted as being associated with COVID-19 [31]. Thirdly, lockdown measures (ban on social gatherings, movement and business restrictions) led to fewer social contacts and thus presumably also to a decrease in social control mechanisms, which resulted in a delay in seeking medical care [32]. All these factors might have contributed to the observation that CAD patients at our institution were in a more critical condition when presenting for CABG surgery.

There was a decline of 14% in CABG surgery in Germany in 2020 compared to 2019 [3]. In the United States, CABG surgery decreased by 36% in 2020 compared to 2019 [13], and in Australia by 10.1% in the first year of the pandemic [15]. In this cohort, CABG surgery decreased by only 2.3% during the pandemic.

Three reasons for this are conceivable. First, this could be partly due to the fact that the federal state of North Rhine-Westphalia, where the UKM is located, was less affected by the pandemic than other regions of Germany [33]. Second, general and visceral surgery numbers were more severely reduced in the first year of the pandemic in Germany (minus 22.7%) [34]. Additionally, a decrease of total patient numbers in the emergency department and a decrease of trauma surgery during the pandemic could be shown for the UKM [35]. As the pandemic situation was a dynamic process, treatment capacities at our institution were reassessed daily and decisions on elective surgery were made considering the daily available ICU-beds for patients undergoing CABG surgery. The reduction in visceral and trauma surgery and the proportionately greater reduction in non-operative and pediatric ICU-beds possibly resulted in an almost constant number of CABG surgery during the pandemic, although this assumption cannot be drawn with certainty from the available data. Third, Germany has a higher supply of ICU beds per population compared to other countries [36], which might mitigate the negative impact of reduced healthcare capacity on ICU capacities for surgical patients.

Additionally, the shift in favor of emergency interventions at the expense of elective surgery during the pandemic probably explains the overall lower decline in CABG surgeries in our study compared to numbers from other regions. Interestingly, indication for cardiac surgery in the whole of Germany was classified as emergency surgery in 11.3% in 2020, which hardly differed from 2019 (10.8%) or 2018 (11.3%) [3].

Two main reasons may have played a role for the higher number of emergency patients during the pandemic in our cohort. Firstly, an expansion of the referral area to the UKM is conceivable, as patients in neighboring regions could not be treated there promptly due to capacity restrictions. Secondly, the patients probably presented at an advanced stage of CAD. The former is less likely, as the distance from the patients’ hometown to the UKM did not differ significantly between the two groups.

Despite worse preoperative cardiac conditions, a higher rate of emergency surgery and longer HLM- and aortic clamping times, the outcome after CABG surgery in the pandemic was comparable, which is unexpected at first sight. After correcting multiple testing, there was no statistically significant difference in postoperative mortality and intraoperative implementation of an ECLS system between both periods. On the other hand, logistic regression showed that patients with urgent and emergency indications for CABG surgery, which was more likely during the pandemic in our cohort, were at higher risk of death. These findings are epidemiologically relevant, as they show a potential increase in morbidity and mortality of CAD patients due to a delayed treatment [11, 17, 37]. Others have shown a dramatic increase in mortality in regions where the number of CABG surgeries were significantly reduced during the pandemic [16, 37].

Our results suggest that not only a decrease in the overall number of CABG surgeries increases the risk for CAD patients, but also delayed treatment, possibly leading to a shift to more emergency procedures, which are then associated with a higher risk of death.

Data from other German heart centers could help to make reliable statements about the consequences of the pandemic in Germany with regard to cardiac surgical care and the outcome of CAD patients [38]. Future multicenter studies over a long observation period are needed to evaluate whether the increase in emergency interventions and more critical preoperative status found in this analysis also applies to other institutions and whether this has a negative impact on the long-term health of CAD patients. This would mean that the treatment of these patients would have to be given even greater priority in future times of crisis with limited resources in the healthcare system.

Several limitations apply to our study. This was a retrospective, monocentric analysis, which means that the results are not easily transferable to the whole of Germany. It is possible that the restrictions at the UKM in the first year of the COVD-19 pandemic were less pronounced than elsewhere. Only basic demographic data was assessed for both groups. It is possible that other baseline characteristics that were not considered in our study design (e.g. non-cardiovascular comorbidities) may have had an impact on outcome data. The long-term outcome was not recorded in this study. Negative effects of a more critical preoperative condition may only become apparent as long-term consequences for cardiovascular health [3]. Limited treatment capacity may also have had an unrecognized negative impact on CAD patients with postponed elective CABG surgery, as these were not included in our study, or on the treatment and outcome of other diseases. As this was an exploratory study that was not based on a formal power analysis, the results can only provide insight into the impact of the pandemic on CAD patients and form the basis for future research into potentially negative effects on outcomes.

## Conclusions

In this study, we analyzed the effects of the COVID-19 pandemic on the cardiac surgical care of patients with CAD at a German university hospital. Overall, the number of CABG surgeries performed during the first year of the pandemic was kept almost constant, although this was accompanied by a prioritization of emergency procedures at the expense of elective surgery. Emergency procedures were associated with a higher risk of inhospital death. Data from other heart centers are needed to determine whether the more critical preoperative conditions and prolonged surgery-times during the pandemic found in this cohort also manifest in a larger population of CAD patients. To get a comprehensive picture of how limited healthcare resources impact different disciplines, interdisciplinary and multicenter research is needed to provide evidence-based prioritization of patients in future pandemics to ensure the least negative impact on all patients.

## Data Availability

The datasets used and/or analysed during the current study are available from the corresponding author on reasonable request.

## Abbreviations

ASA: American society of anesthesiologists
CABG: Coronary artery bypass graft
CAD: Coronary artery disease
ECLS: Extracorporeal life support
HLM: Heart-lung machine
ICU: Intensive care unit
LOS: Length of stay
LVAD: Left ventricular assist device
LVEF: Left ventricular ejection fraction
MANOVA: Multivariate analysis of variance
UKM: University hospital Münster
